# Modeled Impacts of Farming Practices and Structural Agricultural Changes on Nitrogen Fluxes in the Netherlands

**DOI:** 10.1100/tsw.2001.332

**Published:** 2001-11-16

**Authors:** Wim de Vries, Hans Kros, Oene Oenema

**Affiliations:** Alterra Green World Research, Wageningen, The Netherlands

## Abstract

In the Netherlands, nutrient emissions from intensive animal husbandry have contributed to decreased species diversity in (semi) natural terrestrial and aquatic ecosystems, pollution of groundwater, and possibly global warming due to N_2_O emissions. This paper presents the results of a modelling study presenting the impacts of both structural measures and improved farming practices on major nitrogen (N) fluxes, including NH_3_ and N_2_O emission, uptake, leaching, and runoff, in the Netherlands, using input data for the year 2000. Average annual fluxes (Gg N year) for the year 2000 were estimated at 132 for NH_3_ emission (160 Gg NH_3_ year), 28 for N_2_O emission, 50 for N inflow to groundwater, and 15 for N inflow to surface water at a total N input of 1046. At this input, nitrate (NO_3_) concentrations in groundwater often exceeded the target of 50 mg NO_3_ l, specifically in well-drained sandy soils. The ammonia (NH_3_) emissions exceeded emission targets that were set to protect the biodiversity of nonagricultural land. Improved farming practices were calculated to lead to a significant reduction in NH_3_ emissions to the atmosphere and N leaching and runoff to groundwater and surface water, but these improvements were not enough to reach all the targets set for those fluxes. Only strong structural measures clearly improved the situation. The NH_3_ emission target of 30 Gg NH_3_ year, suggested for the year 2030, could not be attained, however, unless pig and poultry farming is completely banned in the Netherlands and all cattle stay almost permanently in low emission stables.

### INTRODUCTION

The rapidly increasing human and animal population and the concomitant request for more food and feed have contributed to a rapid increase in available nitrogen (N) in agriculture[1,2]. Together with N emissions related to industrialisation and increasing traffic, this has led to increased levels of reactive N (all oxidised and reduced forms of N, except for $N_2$) in the environment causing a cascade of effects. These effects include[3,4]:

1. Decreased species diversity and acidification of seminatural terrestrial ecosystems;
2. Eutrophication of surface waters, including excess algal growth and a decrease in species diversity;
3. Pollution of groundwater due to nitrate ($NO_3$) leaching;
4. Global warming due to $N_2O$ emissions; and
5. Air pollution by ozone for which $NO_x$ is a precursor.

The Netherlands is one of the countries with the highest reactive N emissions density in the world. High traffic density and intensive animal husbandry has contributed especially to high oxidised and reduced N emissions per hectare. The estimated total annual N input flux (including biological N fixation) per hectare on agricultural land for the period 1995–1997 is 490 kg for the Netherlands, compared to 142 kg for the European Community[5]. The animal manure production per hectare in the Netherlands is even five times the average European value[5], of which approximately two thirds originates from cattle and one third from pigs and poultry[6]. Fertiliser use is also high in the Netherlands, although animal manure dominates the N input.

With respect to N, most attention in the Netherlands is paid to (1) the adverse effects of excessive N inputs of animal manure, causing excess leaching and runoff to groundwater and surface water, and (2) high emissions of ammonia ($NH_3$) causing adverse effects on nonagricultural ecosystems. To reduce the leaching of $NO_3$ to groundwater and the runoff of N to surface waters, N loss targets have been set, where N loss stands for the difference between inputs and outputs at farm level. Annual N loss targets are 100 kg N $ha^{-1}$ for arable and maize land and 180 kg N $ha^{-1}$ for grassland by the year 2003. For well-drained (dry) sandy soils, those losses are 60 and 140 kg $ha^{-1}$ $year^{-1}$, respectively. These mean N surpluses at farm level have been agreed upon as "satisfactory"[7]. In the Netherlands, a Mineral Accounting System (MINAS) has been introduced as a regulatory policy instrument in agriculture to reach the above-mentioned N loss targets and to fulfill the EU nitrate directive. These targets aim at an N leaching and runoff such that the $NO_3$ concentration in upper groundwater stays below the EU quality criterion of 50 mg $l^{-1}$ and the N concentration in stagnant surface waters below a target of 2.2 mg $l^{-1}$.

Furthermore, the Dutch Ministry of Environment recently set national $NH_3$ emission targets of 100 Gg $NH_3$ $year^{-1}$ for the year 2010, 50 for the year 2020, and 30 for the year 2030, to avoid adverse impacts (specifically in view of biodiversity) on natural ecosystems. In 1995, annual $NH_3$ emissions in the Netherlands were estimated at 146 Gg $NH_3$-N, equivalent to 175 Gg $NH_3$[6], implying a succeeding reduction in $NH_3$ emissions of approximately 45, 70, and 80% compared to this target year.

To determine the effectiveness of policies aimed at the reduction of the $NH_3$ emission, $NO_3$ leaching, and N runoff, it is essential to have information on the fate of N in agricultural soils on a regional and national scale independent of N inputs. In a previous paper[4], information on the fate of N and its inherent uncertainties was presented using an integrated N balance model. This paper presents the results of a modelling study presenting the impacts of both improved farming practices and structural changes in agriculture on N fluxes in the Netherlands, using input data for the year 2000. Improved farming practices include measures to reduce N leaching and runoff to groundwater and surface water, such as reduction of the grazing time, precision fertilisation, optimising of animal feed, improvement of drainage or irrigation, fertiliser reduction, and the use of cover crops. Furthermore, it includes farming practices to reduce N emissions to the air, such as the coverage of manure storage systems and slurry injection. Most of those measures have been taken already, but a clear improvement in those practices is still possible. Furthermore, impacts of structural agricultural changes are calculated including the use of low emission animal housing systems and the reduction of life stock intensity, specifically with respect to pigs and poultry. Similarly, those measures have already partly been taken or are going to be implemented, but strong changes are still possible.

## MODELLING APPROACH AND MODEL APPLICATION

### Modelling Approach

To gain insight into the fate of all major N flows in the Netherlands, a model was developed called INITIATOR (Integrated NITrogen Impact AssessmenT model On a Regional scale), representing all crucial processes in the N chain by simple process descriptions. A flow chart of the considered N inputs and N transformation processes in the model for both terrestrial and aquatic ecosystems is given in Fig. 1.

INITIATOR is a simple N balance model based on empirical linear relationships between the different N fluxes[4]. Linear relationships are very simplistic for biologically mediated processes, but we have chosen a simple approach to maintain transparency, easily gain insight into the overall uncertainty of the N fate in the Netherlands, be able to investigate policy measures by applying the model with available data, and allow inverse modelling. The latter aspect is a unique point of INITIATOR. The model is able to calculate an acceptable N input to the soil or N ceiling in agriculture on the basis of different quality criteria[8], but this aspect is not further discussed in this paper.

In agricultural systems, first the total N input to the soil is calculated as the sum of inputs via animal manure, fertiliser, atmospheric deposition, and biological N fixation. The fate of N in the terrestrial system is calculated as a sequence of occurrences in the order $NH_3$ emissions, followed by N uptake, N accumulation/immobilisation, nitrification, and denitrification in the soil. All N transformation processes are linearly related to the inflow of N (first order kinetics). This implies that $NH_3$ emission due to application depends linearly on the N input into the soil, N uptake on the N input minus the $NH_3$ emission, N immobilisation on the input minus the $NH_3$ emission minus N uptake, etc. The linear transformation constants are a function of type of manure, land use, soil type, and/or hydrological regime. The parameterisation of the equations for estimating the $NH_3$ loss was done in such a way that it included all $NH_3$ losses, including those from animal housing and manure storage systems and from the application of animal manure, fertilisers, and dung and urine from grazing animals to the soil. In the approach, it was implicitly assumed that the manure applied to the soil in a given grid cell (external data) came from the farms in the same grid cell.

The flux of N leaving the terrestrial system is calculated by subtracting all N outputs from the system (emission, uptake, and denitrification) and possibly net accumulation or release from the N inputs to the soil. The leaching loss from the terrestrial systems is partitioned to surface water and to groundwater by multiplying the leaching loss with a runoff fraction (including all pathways for N moving to surface waters) and a leaching fraction (1 - runoff fraction), respectively. Since we are interested in the leaching of N to the groundwater at a 1-m depth below the phreatic level (the depth where $NO_3$ concentrations are measured in the Netherlands), denitrification of N in upper groundwater is

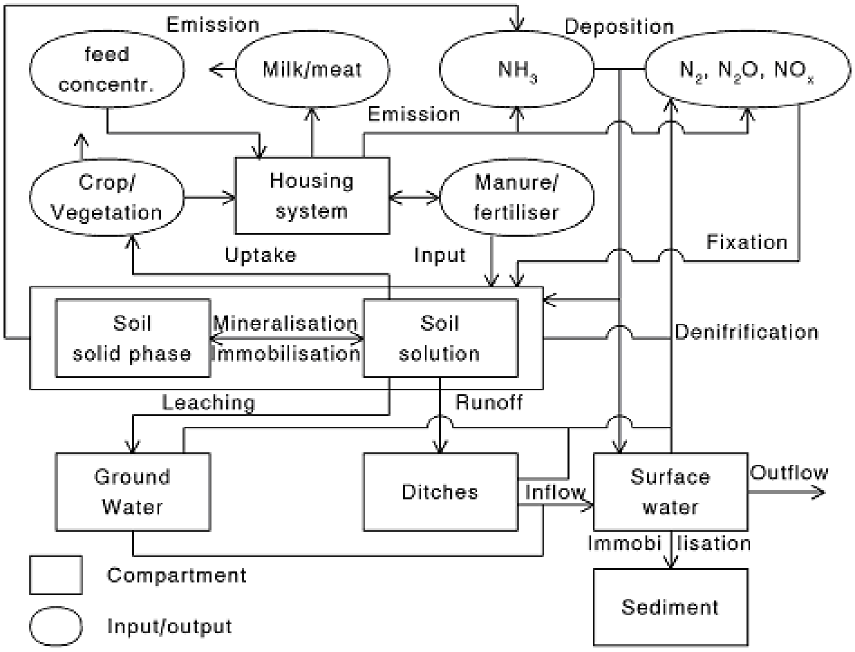

**FIGURE 1**. Overview of the N inputs and processes in terrestrial and aquatic ecosystems.

also considered. The processes considered relevant in aquatic systems are N retention in ditches and larger surface waters, retention being distinguished in denitrification, and accumulation in the sediment. Denitrification is thus calculated in the soil, upper groundwater, ditches, and surface water (compare Fig. 1). We considered runoff from terrestrial systems and direct atmospheric deposition of N to surface waters as input of N in aquatic systems. The various N outputs from soil, groundwater, and surface water and the N retention fluxes in these compartments are calculated with a consistent set of simple equations[4].

### Model Application

The modelling approach was applied to the whole of the Netherlands to gain insight into the impacts of improved farming practices and structural agricultural changes, as described in more detail below, on a national scale. For agriculture, a total number of 2543 plots were distinguished, consisting of a multiple of 500- × 500-m$^2$ grid cells with unique combinations of soil use, soil type, and groundwater table class[9], which determine the parameterisation of N transformation processes. Georeferenced data for the N input via animal manure and fertilisers were based on data statistics at farm and municipal level for the year 2000, using the model CLEAN[10]. Animal manure was divided in cattle, pig, and poultry manure, and in dung and urine deposited on grassland by grazing animals, since this has an influence on the $NH_3$ emissions from either housing and manure storage systems or the soil. N deposition data were based on modelled values at a 1- × 1-km grid scale for the year 2000, using the model DEADM[11]. N fixation was estimated as a function of land use[4].

Estimated total N inputs to agricultural land were slightly larger for animal manure (464 Gg N year$^{-1}$) than for fertiliser (396 Gg N year$^{-1}$). About 66% of the animal manure input originated from cattle, 23% from pigs, and 11% from poultry, including other animals. Cattle manure included the N in excrements from grazing animals, being equal to approximately 35% of the annual N excretion. The estimated input by N deposition and biological N fixation was 90 Gg N year$^{-1}$, leading to a total N input of 950 Gg N year$^{-1}$.

The model parameters describing N transformation processes and transfers were estimated as a function of land use, soil type, and groundwater table class, thus allocating them to combinations occurring in distinct plots. In the agricultural plots, a distinction was made in grassland, maize, and arable land. Soils were divided in sand, loess, clay, and peat. Furthermore, a distinction was made in different hydrological regimes (wetness classes), using groundwater table classes (Gt) from the 1:50,000 soil map with information on the mean highest water level (MHW) used in the plots, according to: (1) wet (poorly drained): MHW <40 cm; (2) moist (moderately drained): MHW 40 to 80 cm; and (3) dry (well drained): MHW >80 cm. Model parameters describing the various N transformations were based on literature data, field observations, results from more detailed model calculations, and expert judgement[4]. An overview of the parameters used in the analyses and the ranges in average values, depending on type of manure, land use, soil type, and hydrology, is given in Table 1.

### Included Measures and their Parameterisation

Measures related to good farming practices, aiming at a more efficient nutrient use, are the coverage of manure storage systems, slurry injection, reduction of grazing time, optimisation of animal feed, precision fertilisation, improvement of drainage or

**TABLE 1**
**Parameters Used in the INITIATOR Model for Agricultural Soils, their Considered Dependence on Land Use, Soil Type and Hydrology and their Overall Ranges[4]**

| Parameter | Explanation | Land Use | Soil Type | Hydrology | Range |
|---|---|---|---|---|---|
| $N_{in,am}$ | N input to the soil via animal manure (kg ha$^{-1}$ year$^{-1}$) | × | — | — | 0–429[a] |
| $N_{in,g}$ | N input to the soil via dung and urine from grazing animals (kg ha$^{-1}$ year$^{-1}$) | × | — | — | 0–172[a] |
| $N_{in,f}$ | N input to the soil via fertiliser (kg ha$^{-1}$ year$^{-1}$) | × | — | — | 14–414[a] |
| $N_{dep}$ | N deposition (kg ha$^{-1}$ year$^{-1}$) | × | — | — | 7–50[a] |
| $N_{fix}$ | Biological N fixation (kg ha$^{-1}$ year$^{-1}$) | × | — | — | 8–25[b] |
| $frNH_{3,em,h}$ | $NH_3$ emission fraction from manure in housing and storage systems (–) | — | — | — | 0.13–0.28[c] |
| $frNH_{3,em,a}$ | $NH_3$ emission fraction from manure applied to land (–) | × | × | — | 0.05–0.10 |
| $frNH_{3,em,g}$ | $NH_3$ emission fraction from dung and urine from grazing animals (–) | — | — | — | 0.08 |
| $frNH_{3,em,f}$ | $NH_3$ emission fraction from fertiliser (–) | — | — | — | 0.02 |
| $N_{up,max}$ | Maximum net N uptake in crops removed from the field (kg ha$^{-1}$ year$^{-1}$) | × | × | × | 110–340 |
| $fr_{up}$ | N uptake fraction (–) | × | — | — | 0.25–0.50[b] |
| $f_{a,am}$ | Factor describing the availability of animal manure relative to fertilisers (–) | — | — | — | 0.6 |
| $frN_{ef}$ | Factor describing the efficiency of N input conversion to animal products (–) | — | — | — | 0.15–0.33[c] |
| $N_{mi,s}$ | Net N mobilisation in peat soils (kg ha$^{-1}$ year$^{-1}$) | × | × | × | 0–400 |
| $fr_{ni,s}$ | Nitrification fraction for the soil (–) | × | × | × | 0.85–0.99 |
| $fr_{de,s}$ | Denitrification fraction for the soil (–) | × | × | × | 0.35–0.94 |
| $fr_{de,gw}$ | Denitrification fraction for upper groundwater (–) | × | × | × | 0.30–0.95 |
| $fr_{de,di}$ | Denitrification fraction for ditches (–) | × | × | × | 0.40–0.94 |
| $fr_{ret}$ | Retention fraction of N input to surface waters (–) | — | — | — | 0.1–0.7[d] |
| $fr_{de,sw}$ | Denitrification fraction compared to the total N retention in surface water (–) | — | — | — | 0.6–1.0[4)] |
| $frNO_{x,ni}$ | Fraction relating total nitrification to $NO_x$ emissions (–) | — | — | — | 0.02 |
| $frNO_{x,de}$ | Fraction relating total denitrification to $NO_x$ emissions (–) | — | — | — | 0.015 |
| $frN_2O_{ni}$ | Fraction relating total nitrification to $NO_x$ emissions (–) | — | × | — | 0.01–0.02 |
| $frN_2O_{de}$ | Fraction relating total denitrification to $NO_x$ emissions (–) | — | × | — | 0.03–0.07 |
| P | Precipitation (mm year$^{-1}$) | | | | 705–874[a] |
| $E_s$ | Soil evaporation (mm year$^{-1}$) | × | — | — | 90–165 |
| $E_t$ | Transpiration (mm year$^{-1}$) | × | × | × | 144–388 |
| $fr_{int}$ | Interception fraction (–) | × | — | — | 0.02–0.12 |
| $fr_{ro}$ | Runoff (lateral flow) fraction (–) | — | × | × | 0.05–0.95 |

[a] These inputs were derived from georeferenced databases, without a direct dependence on land use, soil type, and hydrological regime. Most distinct differences occur for different land use types.
[b] The range equals the range in average values for the different combinations of land use, soil type, and hydrological regime.
[c] The N use efficiency factors and $NH_3$ emission fractions for housing were distinguished by the type of manure (i.e., cattle, pig, and poultry manure).
[d] Retention and denitrification fractions in surface waters were distinguished by the considered geographic region in the Netherlands.

irrigation, fertiliser reduction, and the use of cover crops. Structural changes in agriculture to reduce N inputs include the use of low emission animal housing systems, the reduction of livestock intensity, specifically with respect to pigs and poultry, and the application of manure processing procedures at the farm. The expected impacts of those farming practices and structural measures on the parameters, describing (1) the input of animal manure or fertiliser to the soil, (2) the $NH_3$ emission from housing systems and from manure application, and (3) the net crop uptake, are given in Table 2. The parameterisation of effects is based on Dutch circumstances. Effects related to N emissions are based on Oenema et al.[12], whereas other effects are mainly based on expert judgement. The study should thus be seen as an exploratory analysis to understand what can happen when the mentioned measures are fully implemented.

Covering of manure storage, use of low emission housing systems, and low emission application leads to a reduction in $NH_3$ emission from housing systems and manure application, respectively, whereas manure processing at the farm will increase the emission, but reduce the N input to the soil. The parameterisation of these changes is based on the literature[12]. Reduction of the grazing time implies that the ratio of animal manure produced in the stable and in the meadow changes. The reduction influences the $NH_3$ emission, since this differs in the stable and in the meadow, and the N leaching, since the availability of animal manure differs from dung and urine deposited in the land. Adjustment of animal feeding increases the N use efficiency, implying that more N taken in from feed concentrates and forage (grass, maize) is retained in meat, milk, eggs, etc., thus reducing the N return in manure. It was assumed that the adjustments of feeding would increase the N use efficiency by 20%, causing a reduction in animal manure input at the same N input of about 10%. Precision fertilisation, improvement of drainage or irrigation, and the use of cover crops were all assumed to have an impact on the uptake of N by crops, as given in Table 2. With respect to fertiliser use, we assumed a reduction of the use to an input (in kg ha$^{-1}$ year$^{-1}$) of 60 and 180 for grassland on dry sandy soils and other soils, respectively, and of 55 and 95 for maize and arable land on dry sandy soils and other soils, respectively. Reduction of livestock was set arbitrarily at 50% and was limited to pigs and poultry in this study, whereas manure processing was assumed to take place on all farms.

**TABLE 2**
**Parameterisation of the Impacts of Structural Measures in Agriculture and Good Farming Practices**

| Measures | Dependence | Impacts | Correction Factor |
|---|---|---|---|
| **Farming practices** | | | |
| Cover manure storage | Livestock | $frNH_{3,em,h}$ | Multiply with 0.9[a] |
| Low emission application | Livestock | $frNH_{3,em,a}$ | Multiply with 0.5[a] |
| Reduce grazing time | (Grassland) | $N_{in,am}$; $N_{in,g}$ | Change ratio: add 3/7 of $N_{in,g}$ to $N_{in,am}$ and subtract it from $N_{in,g}$ [b] |
| Adjust animal feeding | Livestock | $N_{in,am}$ | Multiply with 0.9[b] |
| Adjust fertiliser use | Land use | $N_{in,f}$ | Apply according to MINAS[b] |
| Precision fertilisation | Livestock | $f_{a,am}$ | Multiply with 1.25[a] |
| Optimal irrigation | Land use, soil type | $N_{up, max}$ | Change uptake dry soils to moist soils[b] |
| Optimal drainage | Land use, soil type | $N_{up, max}$ | Change uptake wet soils to moist soils[b] |
| Apply cover crops | (Arable land, maize land) | $N_{up,max}$ | Add 40 kg ha$^{-1}$ year$^{-1}$ [b] |
| **Structural measures** | | | |
| Low emission housing | Livestock | | |
| | Cattle | $frNH_{3,em,h}$ | Multiply with 0.35[a] |
| | Pig, poultry | $frNH_{3,em,h}$ | Multiply with 0.2[a] |
| Livestock intensity decrease | Livestock | $N_{in,am}$, $N_{in,pr}$ | Reduction fraction (here 0.5)[c] |
| Manure processing | Livestock | $N_{in,am}$ | Reduction fraction (here 0.5)[c] |
| | | $frNH_{3,em,h}$ | Multiply with 1.05[b] |

[a] Based on literature[12].
[b] Based on expert judgement.
[c] Arbitrary value used in this study to gain insight in a given decrease of livestock intensity or increase in manure processing.

### MODEL RESULTS

Model results first focus on the impacts of farming practices and structural measures on the fluxes of $NH_3$ to the air in view of $NH_3$ emission targets, and on $NO_3$ and N fluxes to groundwater and surface water, as compared to N inputs to the soil. We then discuss the modeled concentrations of $NO_3$ in groundwater and N in surface water in view of critical limits for those concentrations.

#### The Impacts of Farming Practices and Structural Measures on N Emissions to Air, Groundwater, and Surface Water

Model results on the fate of N in the Netherlands for agricultural systems for the year 2000, before and after structural measures in agricultural ecosystems, including optimal farming practices, are given in Table 3. The balances are complete, such that the N production, given in the first row, corrected for the total emissions of $NH_3$, $N_2O$, $NO_x$, and $N_2$, and for the N uptake and accumulation in the soil is equal to the N inflow to groundwater and surface water. Here, N production is defined as the net input of N to the farm (feed concentrates and fertiliser corrected for N output by milk, meat, and crops), while adding the N deposition and biological N fixation. Average annual fluxes (Gg N year$^{-1}$) for the year 2000, using the present N inputs and the estimated values for the parameters influencing the various N processes, were 132 for $NH_3$-N emission (160 Gg $NH_3$), 29 for $N_2O$ emission, 18 for $NO_x$ emission, 50 for N inflow to groundwater, and 15 for N inflow to surface water (Table 3). The difference between these N inputs and outputs is mainly due to an estimated N uptake of 418 Gg N year$^{-1}$ and a total denitrification of 479 Gg N year$^{-1}$. In this calculation, the $N_2O$, $NO_x$, and $N_2$ emission from animal housing systems is also included, but this is calculated to be nearly negligible compared to the emission from soils in accordance to the occurrence of nitrification and denitrification. The total emission of $N_2O$, $NO_x$, and $N_2$ due to (de)nitrification in the housing systems, soil, groundwater, and ditches (in Gg N year$^{-1}$) was calculated at 14, 390, 41, and 34, respectively, showing that most denitrification occurs in the soil[4]. The average N immobilisation was calculated to be negative (a net mineralisation of 48 Gg N year$^{-1}$), following our assumption that N is at steady state in agricultural systems except for peat soils, where net N mineralisation occurs due to decomposition of organic matter. The sum of N uptake and N immobilisation was thus equal to 370 Gg N year$^{-1}$. The assumption that N is at a steady state may also not be true for many grasslands on clay soils.

Using good farming practices leads to an estimated reduction in N input of nearly 20%, an even stronger reduction in $NH_3$ emission (approximately 30%), and nevertheless a slight increase in N uptake, thus causing a strong reduction in N inflow to groundwater (approximately 50%) and surface water (approximately 35%). The reduction in $NH_3$ emission is such that it almost meets the target of 100 Gg $NH_3$ year$^{-1}$ for the year 2010. The reduction

**TABLE 3**
**Calculated Annual Fluxes of N in the Netherlands in the Year 2000 Before and After Measures in Agricultural Ecosystems, Using the INITIATOR Model**

| | N Fluxes (Gg N year$^{-1}$) | | | | | | |
|---|---|---|---|---|---|---|---|
| **Process** | **Standard Run** | **Good Farming Practices** | **Plus Low Emission Housing** | **Plus Reduced Grazing** | **Plus 50% Reduction in Pigs** | **Plus 50% Reduction in Poultry** | **Plus 50% Manure Processing** |
| Production[a] | 1038 | 838 | 838 | 842 | 788 | 760 | 596 |
| $NH_3$ emission[b,c] | 132 (160) | 95 (116) | 50 (61) | 51 (62) | 45 (55) | 43 (52) | 36 (44) |
| $N_2O$ emission[c] | 29 | 20 | 22 | 21 | 19 | 18 | 12 |
| $NO_x$ emission[c] | 18 | 12 | 13 | 13 | 11 | 11 | 7 |
| $N_2$ emission | 425 | 286 | 313 | 307 | 277 | 261 | 165 |
| Uptake[d] | 418 | 437 | 447 | 458 | 448 | 442 | 408 |
| Accumulation | –48 | –48 | –48 | –48 | –48 | –48 | –49 |
| Inflow upper groundwater | 50 | 27 | 30 | 29 | 26 | 24 | 13 |
| Inflow surface water[e] | 15 | 10 | 11 | 11 | 10 | 9 | 6 |

[a] Equals the N input to the farm (feed concentrates and fertiliser corrected for N output by milk, meat, and crops) while adding the N deposition and biological N fixation.
[b] Includes $NH_3$ emission from housing systems and manure application. Values in brackets are given in Gg $NH_3$ year$^{-1}$, to compare them with $NH_3$ emission targets.
[c] Gaseous emissions of $N_2O$, $NO_x$, and $N_2$ are related to nitrification and denitrification in housing systems, soil, upper groundwater, and ditches. The denitrification in large surface waters is not included, but this is relatively small. By far the largest fluxes do occur in the soil[4].
[d] In soils, accumulation is negative, due to net mineralisation in peat soils. The net accumulation in large surface waters is not included, but this is relatively small[4].
[e] The inflow to surface water equals the outflow from all ditches.

in denitrification is associated with a reduction of approximately 30% in $N_2O$ emission, being much larger than the $N_2O$ emission reduction target of 6%.

When using low emission housing systems, the $NH_3$ emission reduces strongly (nearly 50%) to an estimated amount that is close to the target of 50 Gg $NH_3$ $year^{-1}$ for the year 2010. It does, however, increase leaching and runoff by approximately 10%. Combining these approaches with reduced grazing slightly increases the $NH_3$ emission, whereas it slightly reduces leaching and runoff of N to groundwater and surface water. Apparently, the $NH_3$ emission in the stable followed by application is slightly higher than the emission in the meadow, even in the case of using low emission stables. The reduced leaching and runoff is mainly due to a larger N uptake, since the availability of N in animal manure is larger than the N availability in dung and urine deposited in the land. Adding a 50% reduction in pigs leads to an estimated reduction of approximately 10% in both the emission of $NH_3$ and inflow to groundwater and surface water. The same is true for a further 50% reduction in poultry. The limited impact on $NH_3$ emission is because we already assumed that all pigs and poultry do occur in low emission housing systems. Assuming manure processing at 50% of all remaining farms (cattle, pig, and poultry) only slightly decreases the $NH_3$ emission (because manure processing at the farm causes an increase in $NH_3$ emission from the housing system), but it strongly reduces the leaching and runoff of N (Table 3).

### The Impacts of Farming Practices and Structural Measures on the Protection of Groundwater and Surface Water

Despite the relative low inflow of N to upper groundwater and surface water compared to the N input (about 5 and 1.5%, respectively), it does cause an exceedance of critical limits for $NO_3$ in groundwater (50 mg $l^{-1}$) and N in surface water (2.2 mg $l^{-1}$) in large parts of the Netherlands. This is illustrated in Tables 4 and 5 for the standard run. There is a clear decrease in concentrations of $NO_3$ in upper groundwater and of N in ditches in this direction: sand dry > sand moist > loess > clay > peat. The annual average concentration for $NO_3$ in upper groundwater for the whole of the Netherlands was calculated to be exactly equal to the critical limit of 50 mg $l^{-1}$ (Table 4). For the concentration of N in ditches, the value was, however, five times as high as the limit of 2.2 mg $l^{-1}$ (Table 5). The latter limit applies to surface waters, but it is clear that the concentration in surface waters is mainly determined by the inflow from ditches[4]. This result implies that it is much more difficult to reach the limit for surface waters than for groundwater.

Using good farming practices, the average concentration of $NO_3$ and total N reduced by approximately 50% across the whole of the Netherlands. Decreases are both absolutely and relatively largest in the dry sandy soils. Nevertheless, the average concentration in upper groundwater below these soils is calculated to be in excess of the critical limit of 50 mg $l^{-1}$ and the concentration in ditches is still mostly too large. When using low emission housing systems, the situation slightly deteriorates for groundwater and surface water. Reduced grazing causes only a very slight improvement for both groundwater and surface water. Adding a 50% reduction in pigs and a 50% reduction in poultry clearly reduces the $NO_3$ concentrations in upper groundwater, specifically below dry sandy soils, but the average concentration still stays above the critical limit of 50 mg $l^{-1}$. An assumed manure processing at 50% of all the remaining farms, finally, leads to a very strong reduction of both the $NO_3$ concentrations in groundwater and the N concentration in ditches. This strong effect is because cattle farms are dominant in the Netherlands, while pig and poultry farms mainly occur in limited areas with intensive animal husbandry. This leads to average $NO_3$ concentrations in upper groundwater below all soil types that are below the critical limit of 50 mg $l^{-1}$. Considering that the relative uncertainty (defined as the standard deviation divided by the average value times 100%) in calculated concentrations is as high as 50 to 100%, there will still be plots where the $NO_3$ concentration will exceed the critical limit. This area will however be small. For surface water, however, we do calculate an annual average value in excess of the limit of 2.2 mg $l^{-1}$ (Table 5). The $NH_3$ emission in this case still does not meet the ultimate target of 30 Gg

**TABLE 4**
**Calculated Average Area Weighted Concentration of $NO_3$ in Groundwater for Agricultural Land under Different Soil Types Before and After Measures in Agricultural Ecosystems, Using the INITIATOR Model**

| | $NO_3$ Concentration in Upper Groundwater (mg $NO_3$ $l^{-1}$) | | | | | | |
|---|---|---|---|---|---|---|---|
| Soil Type | Standard Run | Good Farming Practices | Plus Low Emission Housing | Plus Reduced Grazing | Plus 50% Reduction in Pigs | Plus 50% Reduction in Poultry | Plus 50% Manure Processing |
| Sand dry | 169 | 71 | 80 | 75 | 66 | 61 | 28 |
| Sand moist/wet | 79 | 59 | 64 | 63 | 60 | 55 | 35 |
| Loess | 69 | 45 | 51 | 50 | 41 | 39 | 22 |
| Clay | 18 | 11 | 12 | 12 | 9.9 | 9.3 | 5.0 |
| Peat | 1.4 | 1.1 | 1.2 | 1.2 | 1.1 | 1.1 | 0.82 |
| All | 50 | 29 | 32 | 31 | 28 | 26 | 15 |

**TABLE 5**
**Calculated Concentration Ranges of N in Ditches for Agricultural Land Under Different Soil Types Before and After Measures in Agricultural Ecosystems, Using the INITIATOR Model**

| | N Concentration in Ditch (mg N/l$^{-1}$) | | | | | | |
|---|---|---|---|---|---|---|---|
| Soil Type | Standard Run | Good Farming Practices | Plus Low Emission Housing | Plus Reduced Grazing | Plus 50% Reduction in Pigs | Plus 50% Reduction in Poultry | Plus 50% Manure Processing |
| Sand dry | 33 | 14 | 16 | 15 | 13 | 12 | 5.4 |
| Sand moist/wet | 17 | 13 | 14 | 14 | 13 | 12 | 7.6 |
| Loess | 16 | 10 | 12 | 11 | 9.4 | 8.9 | 5.1 |
| Clay | 4.4 | 2.6 | 3.0 | 2.9 | 2.5 | 2.3 | 1.3 |
| Peat | 1.6 | 1.3 | 1.4 | 1.4 | 1.3 | 1.2 | 0.93 |
| All | 11 | 6.4 | 7.1 | 6.8 | 6.2 | 5.8 | 3.3 |

$NH_3$ year$^{-1}$ set by the Dutch government for the year 2030 (Table 3).

## DISCUSSION AND CONCLUSIONS

### Results in View of Other Studies

This paper describes results of an integrated analysis using the newly developed INITIATOR model. INITIATOR has not yet been thoroughly validated, but calculated national averages of N fluxes, i.e., $NH_3$ emission, $N_2O$ emission, and total leaching to groundwater, are in line with most previous studies using more sophisticated models[6,13]. Results for the year 1995 are within 5% for most of those fluxes[4]. A comparison of the model results for $NO_3$ concentrations in groundwater with measurements in groundwater below agricultural sandy soils[14] showed that average measured concentrations were approximately 25% lower for grassland, with the exception of wet soils, and 30% higher for arable and maize land[4], but a more thorough site-specific model validation is needed. Regarding the runoff of N to surface water, more attention is needed to validate the results. On a worldwide scale, 21 Tg N is estimated to be transported by rivers to the world's oceans, while N inputs to the watersheds (soils) are about 200 Tg (fertiliser, atmospheric deposition, and manure)[15]. Assuming that a considerable part of the N is denitrified during river transport, this would imply much higher percentages of N inputs to soils ending up in rivers than the 1.5% estimated in this study. Nevertheless, even our low percentages do cause a strong exceedance of the critical limits of 2.2. mg l$^{-1}$, and much higher values are not likely in view of available data on N concentrations in surface waters.

The major difference with previous studies is that they mostly focus on parts of the system (for example, on either $NH_3$ emission, or $N_2O$ emission to the atmosphere, or leaching to groundwater, or runoff to surface waters), but hardly ever on the overall fate of N. Considering this feature of INITIATOR, it is a fast tool to gain initial insight into the impacts of measures in agriculture, which can later be checked more carefully with more detailed models. This paper presents only a preliminary quantification of various measures on N fluxes to air, groundwater, and surface water in view of environmental protection limits, but as such it does give an indication of the direction that is needed to reach those limits.

### Model Results

Results of this study show that the annual $NH_3$ emission and the annual N leaching and runoff to groundwater and surface water for the year 2000 exceed critical limits when improved farming practices or additional structural measures are not implemented. Average annual fluxes (Gg N year$^{-1}$) were estimated at 132 for $NH_3$ emission (160 Gg $NH_3$ year$^{-1}$), 28 for $N_2O$ emission, 50 for N inflow to groundwater, and 15 for N inflow to surface water at a total N input of 1046 Gg N year$^{-1}$. The annual $NH_3$ emission strongly exceeds the emission targets of 100, 50, and 30 Gg $NH_3$ year$^{-1}$ that are set for the years 2010, 2020, and 2030 to protect the biodiversity of nonagricultural land. Both N inflow to groundwater and surface water are relatively low compared to the N input to agricultural soils (on average 5 and 1.5%, respectively). Nevertheless, it does lead to $NO_3$ concentrations in upper groundwater exceeding the EU quality criterion of 50 mg l$^{-1}$ and N concentrations in surface water exceeding quality targets related to eutrophication (2.2 mg l$^{-1}$). This is specifically true in dry sandy soils and, to a lesser extent, also in moist sandy soils and loess soils.

Optimal farming practices in the whole country were calculated to lead to a significant reduction in $NH_3$ emissions to the atmosphere (30%) and N leaching and runoff to groundwater and surface water (approximately 50%). This was, however, not enough to reach the targets set for those fluxes. Only the targeted reduction in emission of nitrous oxides by at least 6% compared to 1995 was easily met by those practices. Only strong structural measures clearly improved the situation. The use of low emission stables, in which all livestock (not only pigs and poultry, but also cows) stay permanently, caused such a strong reduction in $NH_3$ emission and N leaching that the emission target of 50 Gg $NH_3$ year$^{-1}$, set for the year 2020, can nearly be reached, and average $NO_3$ concentrations in upper groundwater even below sandy soils and loess soils approach the critical limit. In surface waters, however, there will still be an excess of N according to the limit set for it.

Application of the most stringent criteria, i.e., full protection of groundwater and surface water and an ultimate $NH_3$ emission of 30 Gg $NH_3$ $year^{-1}$, could not be attained with the measures that were applied in this study. Assuming optimal farming practices, use of low emission animal housing systems in the whole of the Netherlands, with cows nearly permanently in those stables, and a 50% reduction in both pig and poultry farms still causes an $NH_3$ emission of 40 Gg $NH_3$ $year^{-1}$. A complete reduction of pig and poultry farms (not mentioned in this study) would lead to the target of 30 Gg $NH_3$ $year^{-1}$, assuming, however, that the applied $NH_3$ emission reduction of 65% from housing systems with cattle is correct. This is most likely too optimistic, meaning that it will almost be impossible to reach this target by reducing pig and poultry farming only. Furthermore, the N input to the soil from cattle farms may in this situation still lead to N concentrations in surface water in excess of 2.2 mg $l^{-1}$ unless manure processing on the farm takes place.

### Policy Implications

In the Netherlands, policy has so far been focussed on individual environmental issues related to specific problems, including: (1) pollution of groundwater and eutrophication of surface waters (N loss targets), (2) eutrophication and acidification of nature areas ($NH_3$ emission targets), and (3) climate change ($N_2O$ emission targets). This study shows the need to analyse the N problem in the Netherlands in an integrated way, which means that all relevant N fluxes are taken into account simultaneously, preferably in combination with economic impacts and social aspects. The results show that the $N_2O$ target can easily be reached when optimal farming practices are applied, whereas the ultimate $NH_3$ emission target of 30 Gg $NH_3$ $year^{-1}$ for 2030 requires a tremendous reduction in (intensive) animal husbandry in the Netherlands. This difference in ambitions is partly due to the fact that policy targets have not yet been set for individual environmental issues. The most stringent $NH_3$ emission target is based on a 95% protection of natural ecosystems, implying that the expected N depositions do not exceed critical N loads (specifically in view of biodiversity) for 95% of the ecosystems. One can question, however, whether this target will really protect the environment against all adverse impacts of N pollution from agriculture. The relation between this target and the aim of 95% protection is limited due to uncertainties in $NH_3$ emissions, in atmospheric transport, in the contribution of other countries to the reduction in $NH_3$ emission, and in the $NO_x$ emissions both national and abroad. Considering this uncertainty, a balanced consideration of needed reductions in reactive N in agriculture vs. environmental gains for nature is advisable.

| | | |
|---|---|---|
| Received: | August | 8, 2001 |
| Revised: | October | 12, 2001 |
| Accepted: | October | 29, 2001 |
| Published: | November | 16, 2001 |
